# Clinical Effect of Mirikizumab Treatment on Bowel Urgency in Patients with Moderately to Severely Active Ulcerative Colitis and the Clinical Relevance of Bowel Urgency Improvement for Disease Remission

**DOI:** 10.1093/crocol/otac044

**Published:** 2022-12-13

**Authors:** Marla C Dubinsky, David B Clemow, Theresa Hunter Gibble, Xingyuan Li, Severine Vermeire, Tadakazu Hisamatsu, Simon P L Travis

**Affiliations:** Mount Sinai Kravis Children’s Hospital, Mount Sinai, New York, USA; Eli Lilly and Company, Indianapolis, USA; Eli Lilly and Company, Indianapolis, USA; Eli Lilly and Company, Indianapolis, USA; University Hospitals, Leuven, Belgium; Kyorin University School of Medicine, Mitaka, Japan; University of Oxford, Oxford, UK

**Keywords:** bowel urgency, remission, improvement, ulcerative colitis, mirikizumab

## Abstract

**Background:**

Bowel urgency reduces ulcerative colitis patients' quality of life. Mirikizumab, a p19-directed anti-IL-23 antibody, demonstrates ulcerative colitis efficacy. Mirikizumab efficacy to reduce bowel urgency and bowel urgency association with other endpoints were analyzed in 2 Phase 3 trials.

**Methods:**

LUCENT-1 (Induction): 1162 patients randomized 3:1 to intravenous 300 mg mirikizumab or placebo every 4 weeks for 12 weeks. LUCENT-2 (Maintenance): 544 mirikizumab responders during induction were re-randomized 2:1 to subcutaneous mirikizumab 200 mg or placebo every 4 weeks for 40 weeks (52 weeks of continuous treatment). Bowel urgency was measured using the Urgency Numeric Rating Scale (0–10); for patients with LUCENT-1 baseline score ≥3, bowel urgency clinically meaningful improvement (≥3-point decrease) and remission (score ≤1) rates in mirikizumab versus placebo groups were compared at Weeks 12 and 52. Associations between bowel urgency and other efficacy endpoints were assessed at Weeks 12 and 52.

**Results:**

A significantly higher proportion of mirikizumab patients versus placebo achieved clinically meaningful improvement in bowel urgency and remission at Weeks 12 and 52. Significantly higher percentages of patients achieving bowel urgency clinically meaningful improvement or remission, compared with those who did not, also achieved endpoints for clinical, corticosteroid-free, endoscopic, and symptomatic remission; clinical response; normalized fecal calprotectin and C-reactive protein; and improved quality of life.

**Conclusions:**

In patients with ulcerative colitis, bowel urgency improvement was associated with better clinical outcomes than in patients without improvement during induction and maintenance. A greater proportion of mirikizumab patients achieved sustainable bowel urgency improvement and remission compared to placebo patients.

## Graphical Abstract

**Graphical Abstract:**
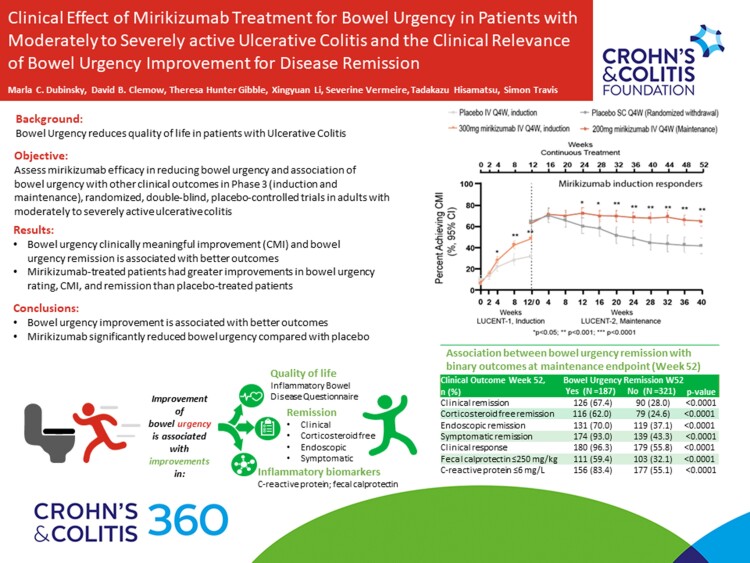

## Introduction

The primary symptoms of ulcerative colitis (UC) include rectal bleeding, increased stool frequency, and bowel movement urgency.^1^ Bowel urgency is the sudden or immediate need to have a bowel movement^2,3^ and is associated with reduced health-related quality of life.^4–6^ Bowel urgency is common in UC and the symptom patients most want to improve.^7–10^ Bowel urgency may persist even when symptoms, such as increased stool frequency and rectal bleeding, are considered inactive.^11^

Despite the importance of bowel urgency to patients, it is often not assessed or prioritized by health-care providers,^9,12–14^ and bowel urgency was not previously a recommended endpoint in clinical trials.^15^ Recently, the United States Food and Drug Administration (FDA) Draft Guidance for UC was updated,^16^ encouraging exploration of additional symptoms of UC identified by patients as important, such as bowel urgency.

Bowel urgency is not included in most UC disease activity indices,^13^ such as the Modified Mayo Score (MMS). To study bowel urgency severity and the effect of mirikizumab on bowel urgency, the Urgency Numeric Rating Scale (UNRS) was developed and psychometrically validated as a novel single-item assessment tool.^17,18^ The UNRS is an 11-point patient-reported scale ranging from 0 (no urgency) to 10 (worst possible urgency) that is used to measure bowel urgency in the past 24 hours. Psychometric evaluation of the UNRS showed clinically meaningful improvement (CMI) is defined as a ≥3-point decrease on the scale, while bowel urgency remission is defined as a score of 0 or 1.^17,18^

Mirikizumab is a p19-targeted IL-23 inhibitor being developed for the treatment of UC.^19,20^ Mirikizumab has demonstrated clinical benefit, including for bowel urgency, in 2 Phase 3 trials in patients with moderately to severely active UC.^19,20^

The current analyses use the UNRS to examine mirikizumab’s efficacy for the treatment of bowel urgency in patients with UC. The association of bowel urgency improvement with other clinical efficacy endpoints, biomarkers, and quality-of-life measures was also examined.

## Methods

Key clinical study methods are noted along with details for the current analyses. The detailed methods for the LUCENT-1 induction and LUCENT-2 maintenance studies have been previously described by D’Haens et al.^19,20^

### Patients and Study Design

The study population for LUCENT-1 included patients (*N* = 1162) between 18 and 80 years of age who had an established diagnosis of UC for at least 3 months prior to baseline and moderately to severely active disease (MMS [4–9]). Participating patients were randomized 3:1 to receive blinded intravenous (IV) administration of 300 mg mirikizumab every 4 weeks (Q4W) with stratification based on their biologic-failed status (yes/no), baseline corticosteroid use (yes/no), baseline disease activity (MMS: [4–6] or [7–9]), and by geographic region (North America/Europe/Other). The biologic-failed patient population (biofailed) was defined as patients who had an inadequate response to, loss of response to, or were intolerant to biologic therapy or the Janus kinase inhibitor tofacitinib for UC.

Patients who had achieved clinical response with blinded mirikizumab treatment (*N* = 544) at the end of the induction period were re-randomized 2:1 to subcutaneous mirikizumab 200 mg (*n* = 365) or placebo Q4W (*n* = 179) for the LUCENT-2 40-week (W) maintenance study. Randomization was stratified by biofailed status (yes/no), corticosteroid use (yes/no) at induction baseline, region (North America/Europe/Other), and induction clinical remission status (yes/no). Patients who lost response to either mirikizumab or placebo after W12 of LUCENT-2 received rescue therapy with 3 doses of open-label 300 mg mirikizumab IV Q4W.

LUCENT-1 and LUCENT-2 together comprised a total of 52 W of continuous treatment, with W12 representing the end of induction and the start (W0) of the 40-week maintenance period.

### Study Endpoints

Table 1 provides a summary of assessment endpoints.

**Table 1.** Endpoint definitions for LUCENT-1 and LUCENT-2.

Endpoint^a^	Definition
Bowel urgency CMI	Change from baseline in UNRS ≥ 3 in patients with UNRS ≥ 3 at induction baseline
Bowel urgency remission	UNRS = 0 or 1 in patients with UNRS ≥ 3 at induction baseline (no or minimal urgency)
Clinical remission	SF = 0 or SF = 1 with ≥1-point decrease in MMS from baseline; RB=0; and ES = 0 or 1 (excluding friability)
Clinical response	≥2-point and ≥30% decrease in MMS from baseline; RB=0 or 1 or, ≥1-point decrease from baseline
Endoscopic remission	ES = 0 or 1 (excluding friability); score ranges 0 to 4; a lower score indicates less mucosal damage
Symptomatic remission	SF = 0 or SF = 1 with ≥1-point decrease in MMS from baseline; RB = 0
Corticosteroid-free remission	Clinical remission at W40, symptomatic remission at W28, and no corticosteroid use for ≥12 weeks prior to W40
IBDQ	Total score ranges from 32 to 224; a higher score indicates a better quality of life
Normal C-reactive protein	≤6 mg/L
Normal fecal calprotectin	≤250 µg/g
UNRS	*Change from baseline in UNRS score; range 0 to 10; a lower score indicates less severe bowel urgency*

Abbreviations: CMI, clinically meaningful improvement; ES, endoscopy subscore; IBDQ, Inflammatory Bowel Disease Questionnaire; MMS, Modified Mayo Score (0–3 for SF, RB, and ES subscores for total 0–9 score); RB, rectal bleeding; SF, stool frequency; UNRS, Urgency Numeric Rating Scale; W, week.

^a^Endpoint analyses for LUCENT-1 were at W12 and for LUCENT-2 at W40 (W52 of continuous treatment).

#### Urgency Numeric Rating Scale

The UNRS is a patient-reported single item that measures the severity of bowel urgency, which is the sudden or immediate need to have a bowel movement, over the past 24 hours using an 11-point scale ranging from 0 (“no urgency”) to 10 (“worst possible urgency”). Patients were provided an electronic diary tool during screening to record information on the severity of bowel urgency on a daily basis. Weekly scores for each patient were calculated by averaging available daily entries over a 7-day period and rounding to the nearest integer. If fewer than 4 days of data were available, then the patient’s data were considered missing for that week.

#### Bowel Urgency Clinical Meaningful Improvement and Bowel Urgency Remission

Bowel urgency Clinical Meaningful Improvement (CMI) is defined as UNRS improvement of ≥3 points in patients with baseline UNRS ≥ 3. Bowel urgency remission is defined as a UNRS score of 0 or 1 (no or minimal urgency) in patients with a baseline UNRS ≥ 3. These thresholds were based on qualitative and psychometric findings by Dubinsky et al.,^17,18,21^ where a UNRS score improvement of ≥3 points was considered to be clinically meaningful for patients with moderately to severely active UC, and that a UNRS score of ≤1 point represented resolution or near resolution of bowel urgency.

#### Inflammatory Bowel Disease Questionnaire

The Inflammatory Bowel Disease Questionnaire (IBDQ) is a 32-item patient-completed questionnaire that measures 4 domains of patients’ lives: symptoms directly related to the primary bowel disturbance, systemic symptoms, emotional function, and social function.^22–24^ Responses are graded on a 7-point Likert scale in which 7 denotes “not a problem at all” and 1 denotes “a very severe problem”. Scores range from 32 to 224; a higher score indicates a better quality of life. Patients recorded their responses to the IBDQ electronically as source data in the tablet device at appropriate visits.

#### Other Clinical Outcomes

Clinical remission was defined as an MMS stool frequency subscore of 0 or 1 with ≥1-point decrease from baseline, a rectal bleeding subscore of 0, and an endoscopic score of 0 or 1, excluding friability. Corticosteroid-free remission was defined as clinical remission at week 40 of maintenance treatment, symptomatic remission at week 28, and no corticosteroid use for ≥12 weeks prior to week 40. Endoscopic remission was defined as an endoscopic score of 0 or 1 (excluding friability) on a 4-point scale: 0 = normal or inactive disease; 1 = mild (erythema, faded vascular pattern); 2 = moderate (marked erythema, loss vascular pattern, erosions, friability); 3 = severe (ulcers or spontaneous bleeding). Symptomatic remission was defined as an MMS stool frequency subscore 0 or 1 with ≥1-point decrease from baseline and a rectal bleeding subscore of 0. Clinical response was defined as ≥2-point and ≥30% decrease in MMS from baseline; rectal bleeding subscore of 0 or 1 or ≥1-point decrease from baseline.

#### Biomarkers

C-reactive protein is an acute-phase protein expressed by hepatocytes in response to inflammatory cytokines, particularly IL-6, TNF, and IL-1β.^25^ In the literature, the normal range for C-reactive protein levels is from ≤3 to ≤8 mg/L.^26^ The current study used ≤6 mg/L to define normal C-reactive protein levels. Fecal calprotectin is a complex consisting of the calcium-binding proteins S100A8 and S100A9, and it is expressed by activated neutrophils or potentially macrophages and monocytes.^25^ Levels of fecal calprotectin in stool correlate with the number of neutrophils in the colorectal mucosa.^25^ As such, fecal calprotectin is used as a biomarker of intestinal inflammation and can be used to predict UC histologic remission^27^ as well as treatment response.^26^ There are variable cutoff points for what are considered normalized levels in the literature, ranging from ≤30 to 250 µg/g, depending on the focus.^26–28^ The present study used ≤250 µg/g to represent normal fecal calprotectin levels.

#### Study Oversight

All patients were required to provide informed consent for participation in the study. The protocol, amendments, and consent documentation were approved by local ethical review boards. The study was registered at the European Network of Centres for Pharmacoepidemiology and Pharmacovigilance and was conducted according to Good Pharmacoepidemiology Practices guidelines and the Declaration of Helsinki.^19^,^20^

#### Statistical Analyses

The analysis population for LUCENT-1 outcomes was the modified intent-to-treat population, which included all randomized patients who received any amount of study treatment.^19^,^20^ The analysis population for LUCENT-2 outcomes through maintenance included the subpopulation of patients who responded to mirikizumab induction therapy at Week 12 (ie mirikizumab induction responders). Unless otherwise specified, baseline values for analyses in LUCENT-2 refer to the values collected at LUCENT-1 baseline.

Treatment comparisons of continuous efficacy and health outcome variables were made using a mixed-effects model for repeated measures (MMRM), with missing at random assumption for handling missing data for LUCENT-1 and 2. Data collected after rescue with study medication in LUCENT-2 were censored in the analysis. Each MMRM model included treatment, baseline value, treatment by visit interactions, baseline value by visit interactions, and stratification factors. Treatment comparison for binary efficacy variables was made using Cochran–Mantel–Haenszel (CMH) tests with missing data considered as nonresponse for LUCENT-1 and LUCENT-2. Data collected after rescue with study medication in LUCENT-2 were also considered missing for the analysis of Week 52 outcome variables. The risk difference and CMH test were both adjusted for the stratification factors.

Association between bowel urgency endpoints (bowel urgency CMI and bowel urgency remission) and dichotomous clinical outcomes (clinical remission, clinical response, symptomatic remission, endoscopic remission, corticosteroid-free remission, normal fecal calprotectin, and normal C-reactive protein) at W12 and W52 were evaluated using Chi-square tests. Odds ratios and nominal 95% confidence intervals were reported. Association between urgency endpoints and IBDQ scores at W12 and W52 was assessed using an analysis of covariance model, with IBDQ score change from baseline as the dependent variable and baseline IBDQ score and CMI or bowel urgency remission as independent variables. Patients were pooled together across mirikizumab and placebo treatment groups for the association analyses. Missing dichotomous clinical outcomes and bowel urgency endpoints were considered as nonresponse, and missing IBDQ scores were imputed using modified baseline observation carried forward.^29^

## Results

### Baseline Demographics and Disease Characteristics

Baseline demographics and disease characteristics were similar between treatment groups in both induction and among the re-randomized mirikizumab induction responders in maintenance (Table 2).

**Table 2.** Baseline demographics and disease characteristics for LUCENT-1 (induction) and LUCENT-2 (maintenance).

Parameter	Induction treatment		Maintenance Treatment Miri Induction Responders	
	Placebo *N* = 294	Mirikizumab 300 mg IV *N* = 868	Placebo *N* = 179	Mirikizumab 200 mg SC *N* = 365
Age, mean years (SD)	41.3 (13.8)	42.9 (13.9)	41.2 (12.8)	43.4 (14.2)
Male, *n* (%)	165 (56)	530 (61)	104 (58)	214 (59)
BMI category, *n* (%)				
• Normal (≥18.5 to <25 kg/m^2^)	149 (51)	451 (52)	97 (54)	196 (54)
• Overweight/obese/extremely obese (≥25 kg/m^2^)	117 (40)	362 (42)	74 (41)	143 (39)
Disease duration, mean years (SD)	6.9 (7.0)	7.2 (6.7)	6.7 (5.6)	6.9 (7.1)
Disease location, *n* (%)				
• Left-sided colitis	188 (64)	544 (63)	119 (66)	234 (64)
• Pancolitis	103 (35)	318 (37)	59 (33)	128 (35)
Modified Mayo Score, *n* (%)				
• Moderate [4–6]	138 (47)	404 (47)	77 (43)	181 (50)
• Severe [7–9]	155 (53)	463 (53)	102 (57)	184 (50)
Mayo endoscopic subscore: severe disease (3), *n* (%)	200 (68)	574 (66)	106 (59)	235 (64)
Bowel urgency severity (UNRS)				
• Median (Q1, Q3)	7.0 (5.0, 8.0)	6.0 (5.0, 8.0)	6.0 (5.0, 8.0)	6.0 (5.0, 8.0)
• UNRS ≥3, *n* (%)	276 (94)	811 (93)	172 (96)	336 (92)
Fecal calprotectin, µg/g, median (Q1, Q3)	1471.5 (626.5, 2944.5)	1559.0 (634.0, 3210.0)	1750.0 (754.0, 3519.0)	1482.0 (558.0, 3045.0)
C-reactive protein (CRP), mg/L, median (Q1, Q3)	4.2 (1.2, 9.5)	4.1 (1.5, 9.6)	3.0 (1.0, 7.7)	3.8 (1.4, 8.7)
IBDQ total score, median (Q1, Q3)	128 (103, 150)	132 (108, 155)	132 (107, 150)	137 (109, 158)
Prior UC therapy, *n* (%)				
• Prior biologic or tofacitinib failure	118 (40)	361 (42)	64 (36)	128 (35)
• Prior anti-TNF failure	97 (33)	325 (37)	58 (32)	112 (31)
• Prior vedolizumab failure	59 (20)	159 (18)	23 (13)	47 (13)
• Prior tofacitinib failure	6 (2)	34 (4)	8 (4)	8 (2)
Number of failed biologics or tofacitinib				
• 0	176 (60)	507 (58)	115 (64)	237 (65)
• 1	65 (22)	180 (21)	35 (20)	77 (21)
• ≥2	53 (18)	181 (21)	29 (16)	51 (14)
Baseline UC therapy, *n* (%)				
• Corticosteroids	113 (38)	351 (40)	68 (38)	135 (37)
• Immunomodulators	69 (23)	211 (24)	39 (22)	78 (21)
• Aminosalicylates	217 (74)	646 (74)	134 (75)	278 (76)

Abbreviations: BMI, body mass index; IBDQ, Inflammatory Bowel Disease Questionnaire; IV, intravenous; N, number of patients; Q, quartile; SC, subcutaneous; SD, standard deviation; TNF, tumor necrosis factor; UC, ulcerative colitis; UNRS, Urgency Numeric Rating Scale.

### Efficacy of Mirikizumab versus Placebo for Bowel Urgency Symptoms

**Figure 1.**
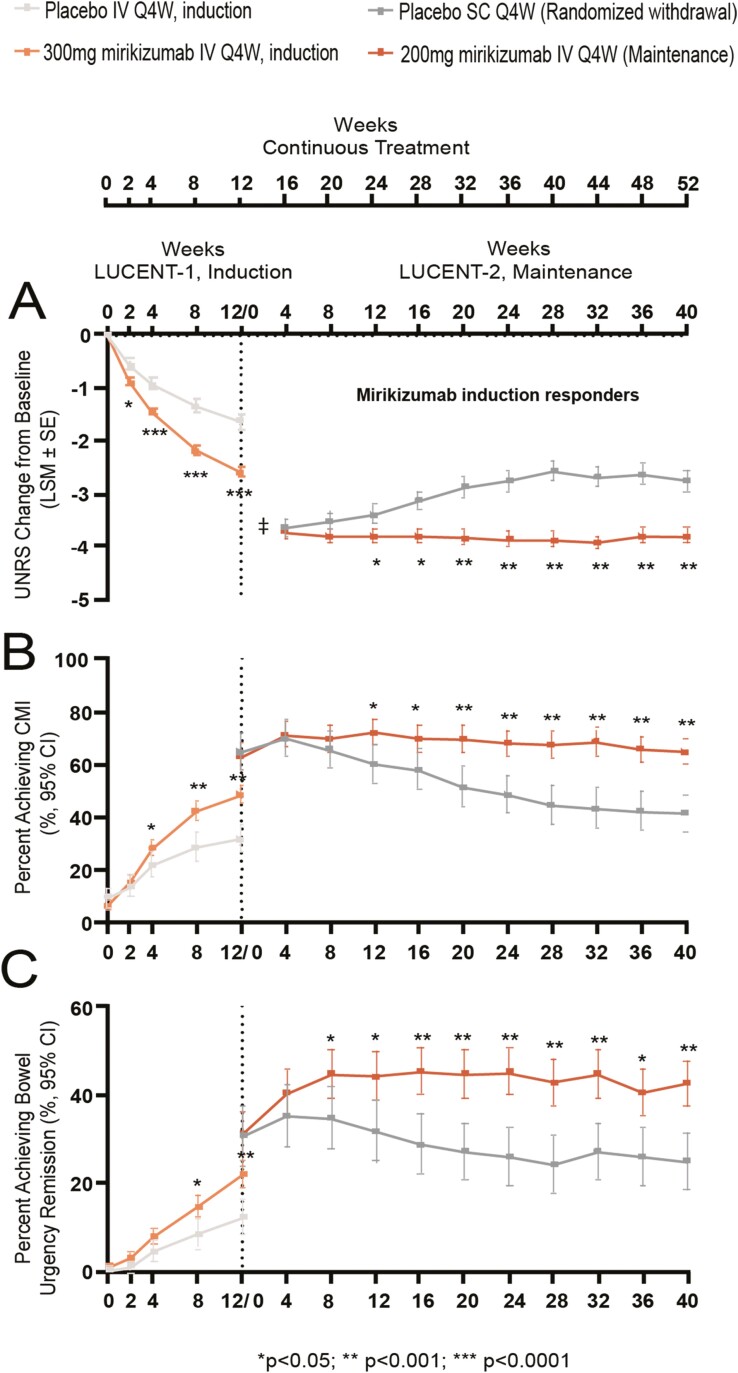
Bowel urgency change from baseline (A), CMI (B), and remission (C) by visit in patients treated with mirikizumab versus placebo over 52 weeks. Abbreviations: CI, confidence interval; CMI, clinically meaningful improvement; IV, intravenous; LSM, least square mean; Q4W, every 4 weeks; SC, subcutaneous; SE, standard error; UNRS, Urgency Numeric Rating Scale. Note: UNRS change from baseline (A) was assessed in the mITT population in LUCENT-1 (mirikizumab: *N* = 868, placebo: *N* = 294) and the subpopulation of mirikizumab induction responders in LUCENT-2 (mirikizumab: *N* = 365, placebo: *N* = 179). Mixed-effects model for repeated measures was used for treatment comparison adjusting for baseline stratification factors. Least squares means were reported for each treatment group except for Week 0 of maintenance (**‡**). Note: Bowel urgency CMI (B) and remission (C) were assessed in the mITT population in patients with UNRS ≥ 3 at induction baseline in LUCENT-1 (mirikizumab: *N* = 811, placebo: *N* = 276) and the subpopulation of mirikizumab induction responders in LUCENT-2 (mirikizumab: *N* = 336, placebo: *N* = 172). Cochran–Mantel–Haenszel (CMH) tests were used for treatment comparison adjusting for baseline stratification factors. Missing data were considered as nonresponse.

Mirikizumab-treated patients reported a significantly greater mean reduction in the UNRS as early as W2 of induction compared to placebo [least square mean (LSM) ± standard error (SE): −0.88 (±0.06) versus -0.57 (±0.10); *p* = 0.004] (Figure 1A). Among mirikizumab induction responders re-randomized to mirikizumab or placebo in the maintenance phase, the mirikizumab treatment group reported a significantly greater mean reduction in UNRS change from induction baseline from W24 (W12 of maintenance) onward compared to placebo [LSM (±SE): −3.81 (±0.13) versus LSM (±SE): −3.39 (±0.18); *p* = 0.034] (Figure 1A). The change from baseline in UNRS remained stable throughout the maintenance period for patients continuing on mirikizumab [W4 of maintenance: −3.73 (±0.12); W40 of maintenance: −3.80 (±0.14)], while patients who were re-randomized to placebo lost some of the improvement gained during induction [W4 of maintenance, LSM(±SE): −3.64 (±0.16); W40 of maintenance, LSM (±SE): −2.74 (±0.20)] (Figure 1A).

Among patients with baseline UNRS greater or equal to 3, a significantly higher proportion of mirikizumab-treated patients versus placebo-treated patients achieved bowel urgency CMI at W12 (48.7% versus 32.2%, *p* < 0.001) and W52 (65.2% versus 41.9%, *p* < 0.001) (Table 3). The improvement was observed from as early as W4 of induction (Figure 1B, *p* = 0.044). In the maintenance phase, among mirikizumab induction responders who were re-randomized to mirikizumab or placebo, treatment group differences were observed from W12 of maintenance (W24 of continuous treatment; *p* = 0.007) and continued through W40 (W52 of continuous treatment; *p* < 0.001) (Figure 1B).

**Table 3.** Assessment of bowel urgency clinically meaningful improvement and remission in patients treated with mirikizumab versus placebo at weeks 12 and 52.

Endpoint	Induction (W12 Analysis)			Maintenance (W52 Analysis)		
	Placebo IV Q4W *N* = 294	300 mg Mirikizumab IV Q4W *N* = 868	*p*-Value^a^	Mirikizumab Induction Responders		
				Placebo SC Q4W *N* = 179	200 mg Mirikizumab SC Q4W *N* = 365	*p*-Value ^a^
UNRS change from baseline (LSM ± SE)						
Overall patients	−1.63 ± 0.14	−2.59 ± 0.08	*p* < 0.001	−2.74 ± 0.20	−3.80 ± 0.14	*p* < 0.001
Biofailed patients^b^	−0.95 ± 0.23	−2.46 ± 0.13	*p* < 0.001	−2.66 ± 0.35	−3.60 ± 0.23	*p* < 0.001
Bowel urgency CMI,^c^*n* (%)						
Overall patients	89/276 (32.2)	395/811 (48.7)	*p* < 0.001	72/172 (41.9)	219/336 (65.2)	*p* < 0.001
Biofailed patients^b^	22/115 (19.1)	157/344 (45.6)	*p* < 0.001	22/63 (34.9)	73/122 (59.8)	*p* = 0.002
Bowel urgency remission,^c^*n* (%)						
Overall patients	34/276 (12.3)	179/811 (22.1)	*p* < 0.001	43/172 (25.0)	144/336 (42.9)	*p* < 0.001
Biofailed patients^b^	5/115 (4.3)	67/344 (19.5)	*p* < 0.001	12/63 (19.0)	43/122 (35.2)	*p* = 0.027

Abbreviations: CMH, Cochran–Mantel–Haenszel; CMI, clinically meaningful improvement; IV, intravenous; LSM, least squares mean; mITT, modified Intent-to-treat population; MMRM, mixed-effects model for repeated measures; N, number of patients; Q4W, every 4 weeks; SC, subcutaneous; SE, standard error; TNF, tumor necrosis factor; UC, ulcerative colitis; UNRS, Urgency Numeric Rating Scale; W, week.

^a^Treatment comparison for UNRS change from baseline was made using MMRM; model included treatment, baseline value, treatment by visit interactions, baseline value by visit interactions, and stratification factors. Treatment comparison for bowel urgency CMI and remission was made using CMH tests adjusting for stratification factors; missing data were considered as nonresponse.

^b^The biofailed patients included patients who had inadequate response to, loss of response to, or were intolerant to a biologic therapy for UC (such as anti-TNFs or anti-integrins) or to the Janus kinase inhibitor tofacitinib.

^c^Bowel urgency CMI and remission were assessed in mITT patients with baseline UNRS ≥3 in LUCENT-1 and mirikizumab induction responders with baseline UNRS ≥3 in LUCENT-2.

Similar to bowel urgency CMI, among patients with baseline UNRS greater or equal to 3, a greater proportion of mirikizumab-treated patients demonstrated bowel urgency remission at W12 (22.1% versus 12.3%, *p* < 0.001) and W52 (42.9% versus 25.0%, *p* < 0.001) compared to placebo (Table 3). In the maintenance phase, mirikizumab induction responders re-randomized to mirikizumab compared to placebo demonstrated significantly higher rates of bowel urgency remission starting at W8 of maintenance (W20 of continuous treatment) compared to placebo (*p* = 0.047) that continued through W40 (W52 of continuous treatment) (Figure 1C). Patients on mirikizumab accrued an additional 13.6% benefit in bowel urgency remission during the first 8 weeks of maintenance therapy (from 31.3% to 44.9%, Figure 1C), while the remission rates declined after W4 of maintenance for placebo-treated patients after randomized mirikizumab withdrawal (Figure 1C).

As shown in Table 3, the efficacy of mirikizumab compared to placebo on bowel urgency improvement was also demonstrated in the subgroup of biofailed patients, defined as patients who had inadequate response to, loss of response to, or were intolerant to a biologic therapy for UC (such as anti-TNFs or anti-integrins) or to the JAK inhibitor tofacitinib. A significantly greater mean reduction in UNRS change from baseline was observed for the mirikizumab group at W12 and W52 (Table 3, both *p* < 0.001). A greater proportion of mirikizumab-treated patients achieved bowel urgency CMI and bowel urgency remission compared to placebo at Week 12 (Table 3, both *p* < 0.001) and W52 (Table 3, *p* = 0.002 for CMI and *p* = 0.027 for urgency remission).

### Associations between Bowel Urgency and Clinical Outcomes at Week 12 and Week 52

**Figure 2.**
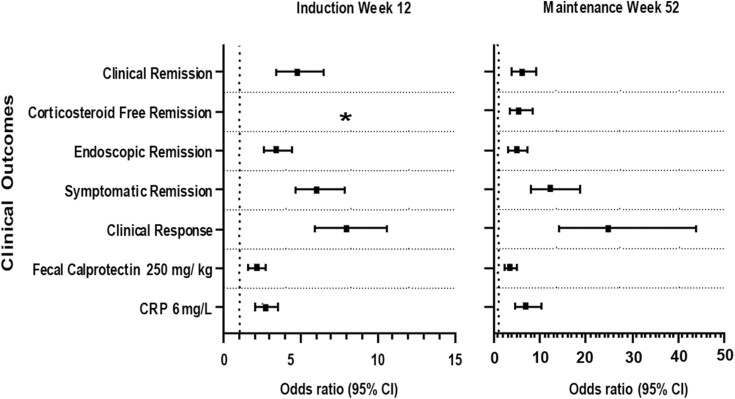
Odds ratio (95% CI) for the association of achieving the clinical outcomes with bowel urgency clinically meaningful improvement (Yes versus No) at induction (W12) and maintenance (W52). Abbreviations: CI, confidence interval; CRP, C-reactive protein; W, week. * Corticosteroid-free remission was not defined in the induction study.

At W12 and W52 (W40 maintenance), patients who achieved bowel urgency CMI had significantly higher rates of achieving all clinical outcomes examined—clinical remission, corticosteroid-free remission (W52 only), endoscopic remission, symptomatic remission, clinical response, normal C-reactive protein, and normal fecal calprotectin—compared with patients not achieving CMI (Table 4, *p*-value <0.0001 for all). The odds ratio of achieving the clinical outcomes and corresponding 95% confidence intervals by bowel urgency CMI status are reported in Figure 2.

**Table 4.** Association between bowel urgency with binary clinical outcomes at induction (W12) and maintenance (W52).

Clinical outcome, *n* (%)	Induction			Maintenance Mirikizumab Induction Responders		
	BU CMI W12 Yes *N* = 484	BU CMI W12 No *N* = 603	*p*-Value ^a^	BU CMI W52 Yes *N* = 291	BU CMI W52 No *N* = 217	*p*-Value^a^
Clinical remission	168 (34.7)	61 (10.1)	<0.0001	174 (59.8)	42 (19.4)	<0.0001
Corticosteroid-free remission	NA^b^	NA^b^	–	157 (54.0)	38 (17.5)	<0.0001
Endoscopic remission	222 (45.9)	121 (20.1)	<0.0001	190 (65.3)	60 (27.6)	<0.0001
Symptomatic remission	310 (64.0)	137 (22.7)	<0.0001	246 (84.5)	67 (30.9)	<0.0001
Clinical response	402 (83.1)	230 (38.1)	<0.0001	274 (94.2)	85 (39.2)	<0.0001
Normal fecal calprotectin (≤250 mg/kg)	200 (41.3)	151 (25.0)	<0.0001	159 (54.6)	55 (25.3)	<0.0001
Normal C-reactive protein (≤6 mg/L)	400 (82.6)	386 (64.0)	<0.0001	242 (83.2)	91 (41.9)	<0.0001

Abbreviations: BU, bowel urgency; CMI, clinically meaningful improvement; N, number of patients; NA, not applicable; W, week.

^a^ *p*-Values were calculated from Chi-square test.

^b^Patients were required to stay on stable dose of corticosteroid during LUCENT-1; therefore, corticosteroid-free remission was not defined in this study.

**Figure 3.**
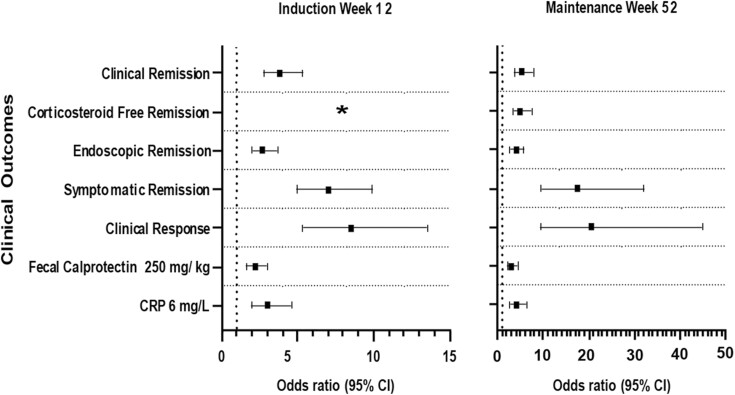
Odds ratio (95% CI) for the association of achieving the clinical outcomes with bowel urgency remission (Yes versus No) at induction (W12) and maintenance (W52). Abbreviations: CI, confidence interval; CRP, C-reactive protein; W, week. * Corticosteroid-free remission was not defined in the induction study.

Similarly, at W12 and W52, patients who achieved bowel urgency remission had significantly higher rates of achieving all clinical outcomes compared to those who did not achieve bowel urgency remission (Table 5, *p*-value <0.0001 for all). Odds ratio of achieving the clinical outcomes and corresponding 95% confidence intervals by bowel urgency remission status are reported in Figure 3.

**Table 5.** Association between bowel urgency remission with binary clinical outcomes at induction (W12) and maintenance (W52).

Clinical outcome, *n* (%)	Induction			Maintenance Mirikizumab Induction Responders		
	BU remission W12 Yes *N* = 213	BU remission W12 No *N* = 874	*p*-Value ^a^	BU remission W52 Yes *N* = 187	BU remission W52 No *N* = 321	*p*-Value ^a^
Clinical remission	90 (42.3)	139 (15.9)	<0.0001	126 (67.4)	90 (28.0)	<0.0001
Corticosteroid-free remission	NA^b^	NA^b^	–	116 (62.0)	79 (24.6)	<0.0001
Endoscopic remission	107 (50.2)	236 (27.0)	<0.0001	131 (70.0)	119 (37.1)	<0.0001
Symptomatic remission	164 (77.0)	283 (32.4)	<0.0001	174 (93.0)	139 (43.3)	<0.0001
Clinical response	191 (89.7)	441 (50.5)	<0.0001	180 (96.3)	179 (55.8)	<0.0001
Fecal calprotectin ≤250 mg/kg	100 (46.9)	251 (28.7)	<0.0001	111 (59.4)	103 (32.1)	<0.0001
C-reactive protein ≤6 mg/L	185 (86.9)	601 (68.8)	<0.0001	156 (83.4)	177 (55.1)	<0.0001

Abbreviations: BU, bowel urgency; N, number of patients; NA, not applicable; W, week.

^a^ *p*-Values were calculated from Chi-square test.

^b^Corticosteroid-free remission was not defined in the induction study.

### Association of Bowel Urgency With Improvement in IBDQ Scores at Week 12 and Week 52

Patients achieving bowel urgency CMI at W12 or W52 (W40 maintenance) had a significantly greater improvement in IBDQ total score and domain scores (bowel symptoms, emotional functions, social functions, and systemic symptoms) than patients not achieving bowel urgency CMI (Table 6, *p* < 0.0001 for all). Similarly, patients achieving bowel urgency remission had significantly greater improvement in IBDQ total and domain scores compared to those who did not achieve bowel urgency remission at W12 or W52 (Table 7, *p* < 0.0001 for all).

**Table 6.** Association between bowel urgency clinically meaningful improvement with IBDQ total and domain scores at W12 and W52.

Induction (W12)				
LSM change from baseline (SE) at W12	BU CMI Yes *N* = 484	BU CMI No *N* = 533	LSM Diff (95% CI)	*p*-Value ^a^
IBDQ total score	52.4 (1.22)	25.6 (1.17)	26.8 (23.5, 30.2)	<0.0001
IBDQ bowel symptoms	19.6 (0.42)	10.2 (0.40)	9.4 (8.3, 10.6)	<0.0001
IBDQ emotional functions	15.9 (0.47)	7.3 (0.45)	8.6 (7.3, 9.9)	<0.0001
IBDQ social functions	9.1 (0.26)	4.4 (0.25)	4.7 (4.0, 5.4)	<0.0001
IBDQ systemic symptoms	7.8 (0.22)	3.8 (0.21)	4.0 (3.4, 4.6)	<0.0001
Maintenance (W52), Mirikizumab Induction Responders				
LSM change from baseline (SE) at W52	BU CMI Yes N=291	BU CMI No N=103	LSM Diff (95% CI)	*p*-Value ^a^
IBDQ total score	62.2 (1.41)	40.4 (2.38)	21.8 (16.3, 27.2)	<0.0001
IBDQ bowel symptoms	22.6 (0.47)	15.4 (0.80)	7.2 (5.4, 9.1)	<0.0001
IBDQ emotional functions	19.5 (0.56)	12.2 (0.94)	7.3 (5.1, 9.4)	<0.0001
IBDQ social functions	10.8 (0.27)	7.4 (0.46)	3.4 (2.3, 4.5)	<0.0001
IBDQ systemic symptoms	9.2 (0.28)	5.6 (0.48)	3.6 (2.5, 4.7)	<0.0001

Abbreviations: BU, bowel urgency; CI, confidence interval; CMI, clinically meaningful improvement; IBDQ, Inflammatory Bowel Disease Questionnaire; LSM, least square mean; LSM Diff, least square mean difference; N, number patients; SE, standard error; W, week.

^a^Treatment comparisons were assessed using an analysis of covariance (ANCOVA) model, with IBDQ score change from baseline as the dependent variable and baseline IBDQ score and BU CMI as independent variables.

**Table 7.** Association between bowel urgency remission with IBDQ total and domain scores at W12 and W52.

Induction (W12)				
LSM change from baseline (SE) at W12	BU remission Yes *N* = 213	BU remission No *N* = 804	LSM Diff (95% CI)	*p*-Value ^a^
IBDQ total score	61.1 (1.91)	32.4 (0.98)	28.7 (24.4, 32.9)	<0.0001
IBDQ bowel symptoms	23.1 (0.64)	12.4 (0.33)	10.6 (9.2, 12.1)	<0.0001
IBDQ emotional functions	18.3 (0.73)	9.6 (0.38)	8.7 (7.1, 10.3)	<0.0001
IBDQ social functions	10.7 (0.40)	5.6 (0.21)	5.1 (4.2, 6.0)	<0.0001
IBDQ systemic symptoms	9.2 (0.34)	4.8 (0.17)	4.4 (3.6, 5.1)	<0.0001
Maintenance (W52), Mirikizumab Induction Responders				
LSM change from baseline (SE) at W 52	BU remission Yes *N* = 187	BU remission No *N* = 207	LSM Diff (95% CI)	*p*-Value ^a^
IBDQ total score	67.8 (1.72)	46.2 (1.65)	21.5 (16.9, 26.2)	<0.0001
IBDQ bowel symptoms	24.6 (0.57)	17.2 (0.55)	7.4 (5.8, 8.9)	<0.0001
IBDQ emotional functions	21.2 (0.69)	14.3 (0.66)	6.8 (5.0, 8.7)	<0.0001
IBDQ social functions	11.8 (0.33)	8.2 (0.32)	3.7 (2.8, 4.6)	<0.0001
IBDQ systemic symptoms	10.2 (0.35)	6.6 (0.33)	3.6 (2.6, 4.5)	<0.0001

Abbreviations: BU, bowel urgency; CI, confidence interval; CMI, clinically meaningful improvement; IBDQ, Inflammatory Bowel Disease Questionnaire; LSM, least square mean; LSM Diff, least square mean difference; N, number patients; SE, standard error; W, week.

^a^Treatment comparisons were assessed using an analysis of covariance (ANCOVA) model, with IBDQ score change from baseline as the dependent variable and baseline IBDQ score and BU remission as independent variables.

The improvement of IBDQ total scores was about 2 times better for patients achieving bowel urgency CMI or remission at induction W12 than those who did not. Among mirikizumab induction responders, the improvements were about 1.5 times better for patients achieving CMI or remission at maintenance W52 (Tables 6 and 7). A similar trend was observed for all 4 domain scores.

## Discussion

Bowel urgency is one of the most troublesome symptoms for patients with ulcerative colitis (UC). The current analyses demonstrate that mirikizumab-treated patients reported greater bowel urgency improvements than placebo-treated patients. Mirikizumab- compared to placebo-treated patients reported a greater mean improvement in UNRS score as early as W2 of induction, and the mirikizumab group had higher proportions of patients achieving bowel urgency CMI at W4 and bowel urgency remission at W8. Among patients responding to mirikizumab induction treatment and re-randomized to mirikizumab or placebo for maintenance treatment, mirikizumab- compared to placebo-treated patients reported a greater mean improvement in UNRS scores and a higher proportion of patients reporting bowel urgency CMI and remission. Mirikizumab-treated patients maintained their improvements through W52.

The superior efficacy in the mirikizumab compared to the placebo treatment group was also demonstrated in the subgroup of patients who had an inadequate response, loss of response, or were intolerant to prior biologic therapy or tofacitinib. This is important because many patients with UC have an inadequate response to, loss of response to, or are intolerant to current advanced therapies, including biologics such as anti-TNF antibodies or anti-integrin antibodies,^30–32^ or tofacitinib. Thus, innovative new therapies are still needed that can help patients dealing with inadequate treatment, including advanced therapies.

Patients, whether randomized to mirikizumab or placebo, who achieved bowel urgency CMI or remission at the end of induction (W12) and at the end of maintenance (W52), had higher rates of achieving clinical outcomes (clinical remission, corticosteroid-free remission, endoscopic remission, symptomatic remission, and clinical response), improved quality of life (IBDQ total and domain scores), and normalized inflammation biomarker values (fecal calprotectin and C-reactive protein). Associations between bowel urgency status and outcomes are important because bowel urgency appears to be an independent predictor distinct from stool frequency and rectal bleeding, which are core to defining symptomatic remission.^33^ This finding provides support for bowel urgency’s current inclusion in clinical indices, guidelines, and future inclusion in definitions of symptomatic and clinical remission.^34–36^

The underlying mechanism(s) resulting in bowel urgency in patients with UC is unclear and probably multifactorial, including inflammatory changes of the rectum, hypersensitivity of the rectum, rectal contractile response/spasms, increased reactivity to rectal distension, increased stool influx due to impaired colon function, and development of submucosal fibrosis associated with chronic inflammation causing deceased rectal wall compliance.^37–45^ Since a combination of mechanisms may be involved in bowel urgency, improvements may not coincide with improvements in other symptoms following treatment. In fact, patients may report bowel urgency even if they are considered in remission based on stool frequency, blood in stool, and endoscopy.^7^ This highlights the clinical relevance of bowel urgency and why assessing severity improvement over time versus a single yes/no time point metric is important. Health-care providers may assume they are addressing bowel urgency when other symptoms improve; however, in practice, approximately 35%–40% of patients with no rectal bleeding or normal stool frequency still have urgency.^7^ Thus, bowel urgency remission may be an important goal independent of other symptoms and regardless of timing in relationship to other symptoms. The UNRS may also be a subjective patient-reported evaluation of a key element of their quality of life independent of other existing clinical parameters.

Previous research investigating the impact of bowel urgency on quality of life and clinical outcomes in patients with UC examined relative association between urgency, stool frequency, and rectal bleeding toward improvement in outcomes with mirikizumab treatment.^33^ For these analyses, the association of urgency status with change in the quality of life was adjusted for stool frequency and rectal bleeding severity. The data demonstrated that the magnitude of correlation was stronger for urgency than stool frequency. Absence of urgency at Week 12 of induction treatment was significantly associated with quality of life at Week 52 of treatment independent from stool frequency or rectal bleeding. These data demonstrate that bowel urgency is an independent clinical outcome to include when assessing UC treatment efficacy. Thus, bowel urgency assessment is important to include in addition to other symptom assessments such as stool frequency and rectal bleeding, even if the response to treatment can also be assessed with other clinical endpoints and the treatment is efficacious across these endpoints.

Importantly, there is evidence of a disconnect between patient and health-care provider perspectives regarding bowel urgency. While studies report that bowel urgency was the second most reported symptom by patients, it was not in the health-care provider-perceived top 3 most reported symptoms.^46^ Health-care providers often do not ask their patients with UC about bowel urgency nor assess its severity or improvement during clinical assessments,^4^ and patients may be too embarrassed to raise the topic.^47^ These data, along with the recognition of bowel urgency in UC clinical guidelines,^34–36,48–51^ indicate the importance of bowel urgency being addressed by health-care providers.

Monitoring bowel urgency can be achieved with an index that includes urgency as a relevant measure of disease activity such as the Simple Clinical Colitis Activity Index.^52^ For those health-care providers who use a UC index such as the SCCAI in their clinic, urgency is being asked about; however, often such indexes are not used clinically, and they can also be prone to bias if the clinician gives an interpretation of the patient’s response.^53^ The SCCAI includes a question on bowel urgency using a four-point scale that lends itself to remote digital monitoring completed by patients.^52^ The United States FDA Draft Guidance^54^ for the development of patient-reported outcomes has been updated to include means of measuring urgency.^16^ These include the Symptoms and Impacts Questionnaire for UC,^55^ Ulcerative Colitis Patient-Reported Outcomes Signs and Symptoms diary,^14^, and the UNRS.^17,18,21^ The UNRS is easily understood by patients,^56^ has been confirmed by patients to include appropriate response options,^17,18,21^ and has demonstrated construct validity and reliability.^17,18,21^ As a single-item nonredundant measurement of other symptoms, the UNRS does not rely on specific descriptors that may not be relevant to all patients due to the varied personal experiences of bowel urgency.^21,56^ Importantly, the UNRS moves beyond yes/no data to address the severity and examine improvement over time in more detail.^56^ For these reasons, the UNRS was used for the current analyses.

For an NRS-based patient-reported outcome measure to inform clinical trials or clinical practice, the scores and score changes must be interpretable. A psychometric study found that regardless of UNRS score severity starting point (3 to 10), a UNRS score improvement of ≥3 points was a CMI for patients with moderately to severely active UC and that a UNRS score of ≤1 point represents bowel urgency remission.^18^ A UNRS score of 0 is defined as “no urgency,” however, some level of variability is expected since bowel urgency can occur in healthy people without underlying inflammation.^57^ These data support that patients in remission or with inactive disease may still report minimal levels of bowel urgency they consider “normal.” Thus, achieving a mean score of 0 on the 11-point UNRS scale may be an unrealistic treatment target, which is why UNRS 0 or 1 was used for remission.

A limitation of this study is that the maintenance population only included responders from the induction period. The data were furthermore self-reported by patients, which while valuable may be less robust than some more objective measurements. Additional research is needed regarding the specific relevance of individual values across the 0 to 10-point UNRS as well as mild versus moderate or severe score categorization and the impact of the absolute value of the UNRS on long-term prognosis. Future research should include bowel urgency improvement predictiveness of downstream clinical outcomes as well as relative contribution compared to stool frequency and rectal bleeding. This research should include an examination of Phase 3 datasets to further examine bowel urgency as an independent factor for the assessment of UC therapy response. Additionally, elucidation of timing and relationship of treatment effect on bowel urgency in comparison to other clinical markers is needed to gain further understanding of the association of bowel urgency with other clinical outcomes, including endoscopy and histology, and how bowel urgency improvement may or may not coincide with stool frequency or rectal bleeding. Whether clinicians should implement treatment intensification with the aim to achieve bowel urgency remission when other symptoms are controlled may need additional investigation, particularly regarding the long-term management of UC.

## Conclusions

Bowel urgency is one of the most disruptive symptoms for patients with UC. The new UNRS assessment tool was able to quantify the baseline level and change in bowel urgency after UC treatment. For patients with moderately to severely active UC, mirikizumab-treated patients reported greater statistically significant improvements in UNRS scores, the proportion of patients achieving bowel urgency CMI, and patients achieving bowel urgency remission compared with placebo-treated patients for both the induction and maintenance treatment periods. Bowel urgency CMI or remission was associated with better outcomes during induction and maintenance treatment for both mirikizumab and placebo patients: clinical outcomes, including clinical remission, corticosteroid-free remission, endoscopic remission, symptomatic remission, and clinical response; quality of life as assessed by the IBDQ, including total score and domain scores for bowel symptoms, emotional functions, social functions, and systemic symptoms; and the inflammation biomarkers fecal calprotectin and C-reactive protein.

## Data Availability

Data are available on reasonable request. Lilly provides access to all individual participant data collected during the trial, after anonymization. Data are available to request after primary publication acceptance. No expiration date for data requests is currently set once data are made available. Access is provided after a proposal has been approved by an independent review committee identified for this purpose and after receipt of a signed data-sharing agreement. Data and documents, including the study protocol, statistical analysis plan, clinical study report, blank or annotated case report forms, will be provided in a secure data-sharing environment. For details on submitting a request, see the instructions provided at https://vivli.org.

## Clinical Studies

LUCENT-1=NCT03518086; LUCENT-2=NCT03524092.
